# Wild at heart: 34-year-old male with new onset dyspnea, heart failure and history of amphetamine use; a case report

**DOI:** 10.1186/s43044-019-0026-y

**Published:** 2019-10-28

**Authors:** Hossein Navid, Hamidreza Soleimani, Kaveh Hosseini

**Affiliations:** 1 Department of Heart Failure and Heart Transplantation, Tehran Heart Center, Tehran University of Medical Sciences, Tehran, Iran; 2 Tehran Heart Center, Tehran University of Medical Sciences, North Karegar Avenue, Tehran, Iran

**Keywords:** Spontaneous coronary artery dissection, Ischemic cardiomyopathy, Methamphetamine abuse

## Abstract

**Background:**

Spontaneous coronary artery dissection (SCAD) is a rather rare cause of acute coronary syndrome with a preponderance for young female patients. Amphetamines are now the second most widely used substance drugs in the world and they are associated with a myriad of cardiac diseases including cardiomyopathies and SCADs. There is much uncertainty regarding the best treatment strategy in such cases and decision-making remains mostly individualized and based on expert opinions.

**Case presentation:**

A 34-year-old male with an unremarkable past medical history presented to a cardiologist with prominent dyspnea and orthopnea. He reported occasional methamphetamine use from 3 years before the presentation. An echocardiogram showed an enlarged left ventricle and severe systolic dysfunction with an ejection fraction of 10–15%. Coronary angiography revealed multiple linear dissections in both left anterior descending coronary artery (LAD) and left circumflex coronary artery (LCX). The patient’s right coronary artery (RCA) showed occlusion in the proximal segment. The patient was diagnosed with amphetamine-induced spontaneous coronary artery dissection and resultant ischemic cardiomyopathy. After thorough evaluation, medical treatment ensued.

**Conclusions:**

Methamphetamine abusers have a 3.7 fold risk of developing some form of a cardiomyopathy in comparison to individuals without amphetamine abuse. Coronary artery dissection and increased thrombus burden are some of the mechanisms responsible for ischemic cardiomyopathy in these groups of patients.

## Background

Spontaneous coronary artery dissection (SCAD) is a rather rare cause of acute coronary syndrome (ACS) with a preponderance for young female patients [1]. Up until this point, less than 800 cases of SCAD have been reported in literature since 1931 [2] and while most of these reports have focused on SCAD’s ACS and sudden cardiac death (SCD) presentations it is obvious that the real numbers are much higher, the reason for this underdiagnose being SCAD’s challenging and often disparate symptoms in young patients with otherwise no coronary artery disease risk factors [3, 4]. Amphetamines are now the second most widely used substance drugs in the world and they are associated with a myriad of cardiac diseases from hypertension to tachycardia to fatal arrhythmias to coronary, carotid, and aortic dissections to cardiomyopathies to death [5]. There are case reports of amphetamine and its derivatives being responsible for coronary dissections [2]. Here, we present a 34-year-old male patient with severe dyspnea and multiple coronary dissections on coronary angiogram who was diagnosed with amphetamine-induced SCAD which had resulted in ischemic cardiomyopathy.

## Case presentation

A 34-year-old male with an unremarkable past medical history and negative family history presented with prominent dyspnea and orthopnea from 6 months before, his New York Heart Association (NYHA) functional class was III, he was a current cigarette smoker, and he reported methamphetamine use from 3 years before presentation. An echocardiogram revealed an enlarged left ventricle (LV) and severe systolic dysfunction with an ejection fraction (EF) of 10–15%. He was referred for consideration for heart transplant.

**Fig. 1:**
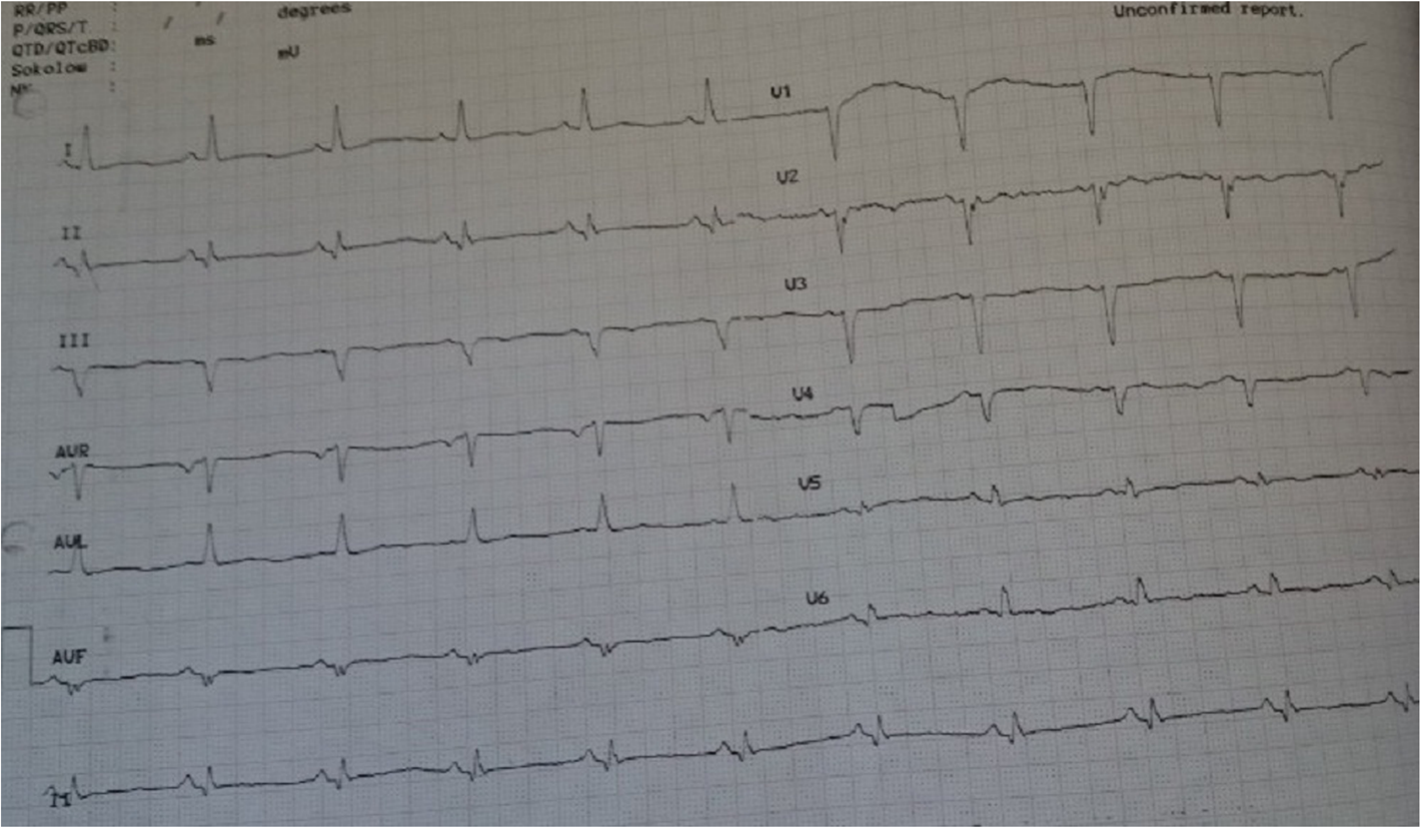
The patient’s electrocardiogram showing a normal sinus rhythm, non-specific ST segment, and T wave changes plus poor R wave progression in limb leads

On physical examination, he appeared pale, his jugular venous pressure seemed elevated, there were bibasilar rales in lung auscultation, and his legs were edematous. He reported atypical chest pain with no relationship to exertion and emphasized that his biggest problem is his dyspnea and “inability to sleep.” He underwent another echocardiography which confirmed the previous echocardiogram’s findings of severe LV dilation with severe systolic dysfunction, LVEF = 10–15%. Smokey pattern was observed in LV, and the apex of LV was reported to have no visible clot. Right ventricular size was increased and its function was mildly reduced. There were no significant valvular findings. An electrocardiogram showed normal sinus rhythm, non-specific ST segment and T wave changes along with poor R wave progression in precordial leads (Fig. 1). His lab tests are shown in Table 1.

**Table 1:** Lab Test Results

Test	Result
White blood cell	6300/cum^3^
Hemoglobin	11.02 g/dL
Platelet	145,200/cum^3^
Creatinine	1.1 mg/dL
Potassium	4.0 meq/l
Sodium	139 meq/l
FBS	77 mg/dL
Triglyceride	95 mg/dL
Cholesterol	127 mg/dL
Low-density lipid	32 mg/dL
High-density lipid	75 mg/dL
AST	20 IU/L
ALT	23 IU/L
Alkaline phosphatase	314 IU/L
Total bilirubin	0.69 mg/dL
Direct bilirubin	0.24 mg/dL
Ferritin	377.3 ng/mL
Serum iron	101 micg/mL
TIBC	251 micg/mL
T3	1.13 ng/mL (0.58–1.59)
T4	10.0 micg/dL (5.1–14.1)
TSH	2.62 uIU (mL) (0.35–4.94)

**Fig. 2:**
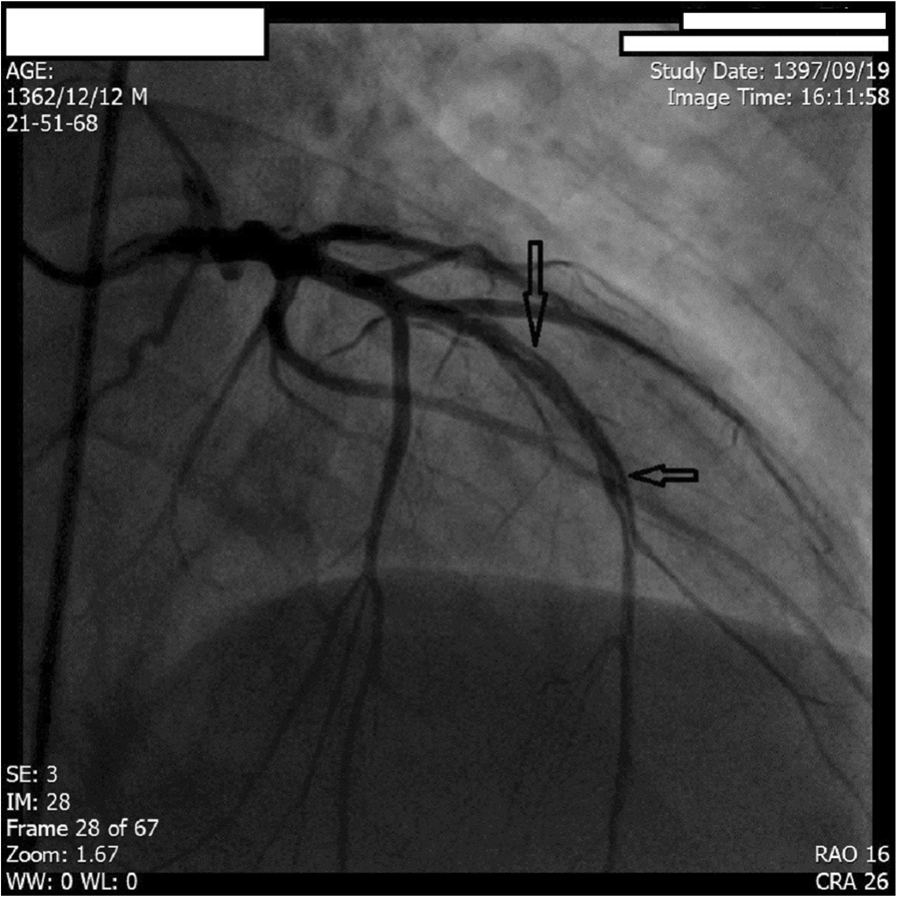
Coronary angiogram showing linear dissection flap in left anterior descending artery (arrows)

**Fig. 3:**
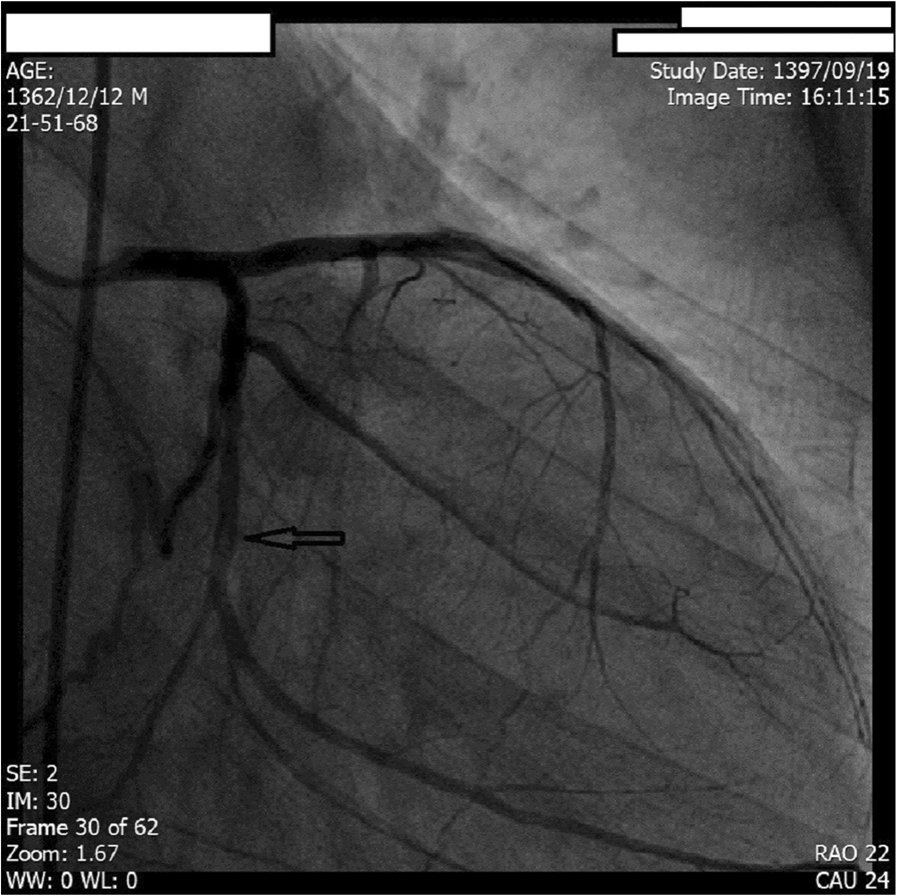
Coronary angiography showing dissection flap in left circumflex coronary artery (arrow)

**Fig. 4:**
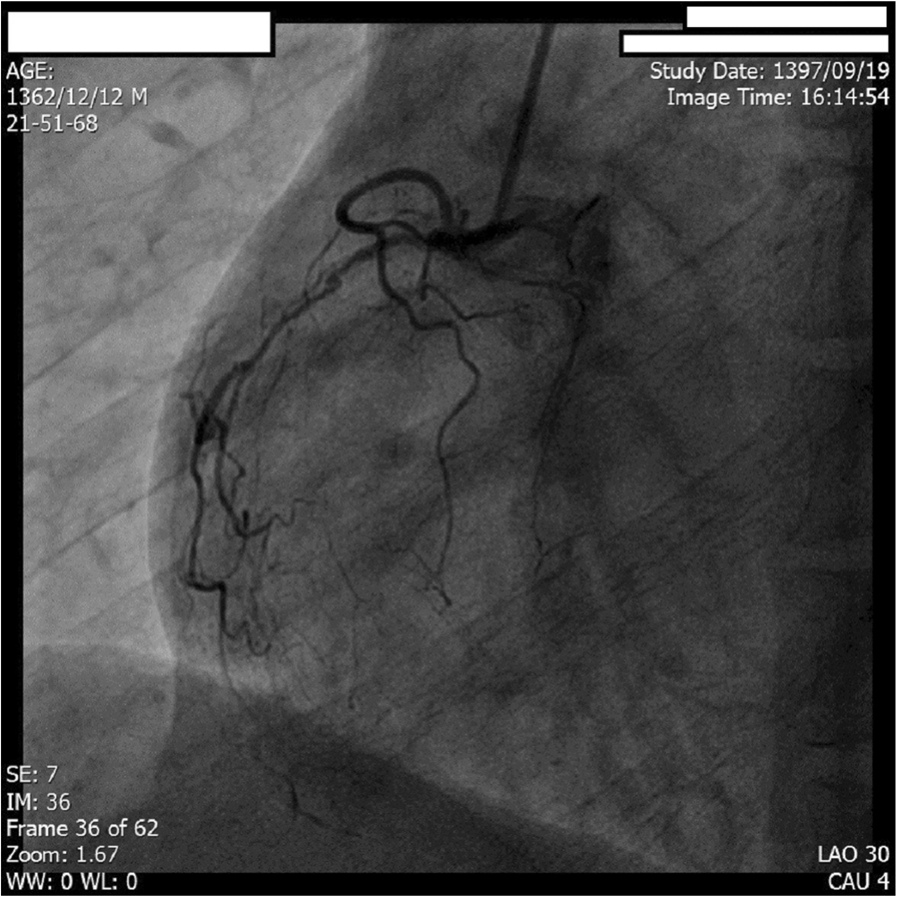
Coronary angiography showing a totally occluded right coronary artery from the proximal portion

Coronary arteriography was scheduled to assess coronary anatomy to rule out possible ischemic cardiomyopathy. Angiography revealed multiple linear dissections in both left anterior descending coronary artery (LAD) and left circumflex coronary artery (LCX). The patient’s right coronary artery (RCA) was occluded in the proximal segment. The patient was diagnosed with amphetamine-induced spontaneous coronary artery dissection with the resulting coronary artery disease being responsible for his LV dysfunction, thus receiving a diagnosis of ischemic cardiomyopathy caused by distal coronary blood flow impairment due to coronary artery dissection flaps (Figs. 2, 3, and 4).

The patient did not have any of the traditional risk factors of atherosclerotic coronary artery disease (CAD) which makes a diagnosis of pure atherosclerotic CAD less likely. Visible dissection flaps in left system further reinforces the idea that it was indeed the dissection flaps which caused distal coronary blood flow impairment. In the right system, the appearance of coronary artery is mostly compatible with type III of coronary artery dissection; this type mimics atherosclerotic disease most and its differentiation from a true atherosclerotic lesion is often challenging. Features supporting a diagnosis of SCAD are lack of atherosclerotic lesions in other coronary vascular beds, long lesions, hazy lesions, and linear stenosis. The patient’s age and set of risk factors can also help in this crucial differentiation.

Myocardial perfusion imaging (MPI) for evaluation of the viability of myocardium was performed in the following week and more than 70% of entire myocardium (including most of the anterior segments and the entire inferior segments) were reported to be non-viable. Consultation with an interventional cardiologist was done and given that in our patient MPI results indicated a vast amount of myocardium as non-viable it was deemed unnecessary to pursue a revascularization approach so after extensive consultations with our Heart Team Guideline Directed Medical Therapy (GDMT) was advised with close follow-up. He was discharged with Aspirin 80 mg daily, Clopidogrel 75 mg daily, Losartan 25 mg daily, Folic Acid 1 mg daily, Spironolactone 25 mg daily, and Furosemide 20 mg TDS. A meeting with a psychiatrist specialized in the management of drug abuse was scheduled.

One remaining aspect of our patient’s management was decision-making regarding heart transplantation. It is widely accepted that heart transplantation is contraindicated in active substance abusers because of the increased risk for relapse and graft compromise. Given these concerns and the fact that donors and suitable organs are unfortunately not easily accessible in Iran, we decided to exclude this treatment option for this patient due to his active substance abuse and continue with medical therapy.

There are many differential diagnoses for a patient with dyspnea and reduced ejection fraction. While a wide spectrum of diseases with various etiologies (ischemic, drug-induced, metabolic, hereditary, and systemic diseases) [6] can cause heart failure with reduced ejection fraction (HFrEF) but ischemic cardiomyopathy remains the most common reason. Numerous drugs (anticancer agents, immunomodulating, antidiabetic, antipsychotic drugs, appetite suppressants, antibiotics, and antifungals to name a few) can adversely affect the heart and its pumping features leading to the development or worsening of heart failure [7].

Reports of cardiotoxic effects of amphetamine were first noted in the 1970s with Rajis describing various pathologic lesions like cardiac chamber enlargement, left ventricular hypertrophy, hemorrhage, and fibrosis in a post mortem analysis of 14 subjects of methamphetamine abuse [8]. In 1989, the first case report of a patient with left ventricular dysfunction primarily due to methamphetamine abuse was published [9].

It has been postulated that methamphetamine abusers have a 3.7 fold risk of developing cardiomyopathy in comparison to individuals without amphetamine abuse [10]. Hypertension, acute coronary syndromes, pulmonary arterial hypertension, and aortic dissections are other known complications of methamphetamine abuse [11]. Sympathomimetic effects of amphetamines can increase the heart rate and blood pressure [12] and coronary artery spasm has been proposed as a mechanism for myocardial ischemia and infarction in patients who abuse amphetamine [13]. Increased thrombus burden has been noted as an underlying reason for acute coronary syndromes is amphetamine abusers [14].

While there are known risk factors for the development of spontaneous coronary dissection like one being in the post-partum period, fibromuscular dysplasia (FMD), connective tissue diseases, and hormonal therapy [15], there are only a handful of case reports describing a causative relationship between amphetamine abuse and coronary artery dissections [16].

Due to the condition being rare and its presentations being non-specific, the optimal therapeutic approach is not yet clear. Choosing medical therapy or interventional or surgical revascularization approaches should be individualized and based on patient’s characteristics, the frequency, and severity of symptoms and extent of myocardium at risk.

While treatment with aspirin, clopidogrel, and beta-blockers have been proposed in cases of single-coronary artery dissection with little myocardium at risk and hemodynamic stability [17], the course of action in a patient like ours with extensive myocardial damage and clinical symptoms of heart failure is less known. In this case, we decided to treat the patient with routine heart failure medications (including diuretics, angiotensin receptor blockers, and mineralocorticoid receptor antagonists) and a dual antiplatelet therapy for at least 1 year.

There are reports that substance abusers can undergo successful transplantation but they are still at greater risk of subsequent complications, dug non-compliance, and eventual graft failure [18]. Authors of this report propose that a multidisciplinary team including cardiologists, psychiatrists, medical ethics specialist, and social workers assess the individual’s medical, social, and economic features and then decide to whether nominate the patient for receiving transplant or not. It must be noted that donor hearts are not easily found in most parts of the world and nominating patients with history of substance abuse for a heart transplant—or any other transplant for that matter—must be addressed with utter discretion.

## Conclusions

SCAD is a condition that is getting more recognized by each passing day, and while most of the attention is directed towards recognizing it in a timely fashion in acute settings, it must be kept in mind that there are chronic cases of SCAD which might lead to cardiovascular symptoms. It is also prudent for cardiologists to be aware of the detrimental effects of amphetamines on cardiac function and counsel their patients. Nominating patients with history of substance abuse for heart transplant is considered a contraindication in most academic cycles but an individualized approach in situations like this is advised.

## Data Availability

All the data and corresponding material (including electrocardiography, echocardiography, lab results, angiographic, and MPI results) of this case report are available on request from the corresponding author. The data are not publicly available due to restrictions regarding patient privacy.

## Abbreviations

ACS: Acute coronary syndrome
EF: Ejection fraction
FMD: Fibro muscular dysplasia
GDMT: Guideline directed medical therapy
HFrEF: Heart Failure with Reduced Ejection Fraction
LAD: Left anterior descending coronary artery
LCX: Left circumflex coronary artery
LV: Left ventricle
MPI: Myocardial perfusion imaging
NYHA: New York Heart Association
RCA: Right coronary artery
SCAD: Spontaneous coronary artery dissection
SCD: Sudden cardiac death
